# Therapeutic and Transmission-Blocking  Efficacy of Dihydroartemisinin/Piperaquine and Chloroquine against *Plasmodium vivax* Malaria, Cambodia

**DOI:** 10.3201/eid2408.170768

**Published:** 2018-08

**Authors:** Jean Popovici, Amelie Vantaux, Lyse Primault, Reingsey Samreth, Eak Por Piv, Sophalai Bin, Saorin Kim, Dysoley Lek, David Serre, Didier Menard

**Affiliations:** Institut Pasteur, Phnom Penh, Cambodia (J. Popovici, A. Vantaux, L. Primault, R. Samreth, E.P. Piv, S. Bin, S. Kim, D. Menard);; National Center for Malaria Control, Phnom Penh (D. Lek);; University of Maryland School of Medicine, Baltimore, Maryland, USA (D. Serre);; Institut Pasteur, Paris, France (D. Menard)

**Keywords:** malaria, Plasmodium vivax, parasites, chloroquine, dihydroartemisinin/piperaquine, DHA/PPQ, therapeutic efficacy, transmission-blocking efficacy, efficacy, transmission, Anopheles dirus, mosquitoes, infectivity, feeding assays, Cambodia

## Abstract

We assessed the efficacy of standard 3-day courses of chloroquine and dihydroartemisinin/piperaquine against *Plasmodium vivax* malaria. Compared with chloroquine, dihydroartemisinin/piperaquine was faster in clearing asexual *P. vivax* parasites and blocking human-to-mosquito transmission. This drug combination was also more effective in preventing potential recurrences for >2 months.

*Plasmodium vivax* is the most widespread human malaria parasite. Almost 2.5 billion persons are at risk for infection in >90 countries (*1*,*2*). Since the 1950s–1960s, Southeast Asia has been the cradle of emergence and spread of *P. falciparum* antimalarial drug resistance, a major obstacle for malaria control. Over the past decade, control efforts in Cambodia have led to an impressive decrease in malaria burden, with a slower decrease of *P. vivax* than for *P. falciparum* (*3*).

*P. vivax* resistance to chloroquine has emerged more recently; the first cases were observed in 2009 in Rattanakiri Province in northeastern Cambodia (17.4% treatment failures after 28 days of follow-up), which led to withdrawal of chloroquine and use of dihydroartemisinin/piperaquine (DHA/PPQ) as first-line therapy for uncomplicated *P. vivax* malaria in 2012 (*4*). We assessed the efficacy of standard 3-day courses of chloroquine and DHA/PPQ for treating *P. vivax* malaria, preventing recurrences, and blocking human-to-mosquito transmission.

## The Study

We conducted an open-label, randomized, control trial in June–December 2014 in Rattanakiri Province, Cambodia. Febrile patients or patients with a history of fever in the previous 48 h who sought treatment in health facilities and had positive results by rapid diagnostic test (CareStart Malaria HRP2/pLDH Pf/PAN Combo; Access Bio, Inc., Somerset, NJ, USA) for non–*P. falciparum* malaria were offered participation in the study. Pregnant or lactating women and patients with signs of severe malaria, other known illnesses, or inability to provide informed consent were excluded. Patients with *P. vivax* monoinfection confirmed by PCR were eligible for the study (*5*). 

At enrollment, after we obtained written informed consent, patients were randomized to receive supervised standard 3-day courses of DHA/PPQ (Duo-Cotecxin; Zhejiang Holley Nanhu Pharmaceutical Co., Ltd., Jiaxing, China) or chloroquine (Nivaquine; Sanofi-Aventis, Paris, France). For each participant, medical histories were obtained and clinical and biological examinations performed. We followed up with patients according to an extended World Health Organization protocol on days 1, 2, 3, 5, and 7 and then weekly until day 63. At each visit, we performed clinical examinations and obtained an axillary temperature and a capillary blood sample. 

Malaria parasites were detected by microscopy (Giemsa-stained blood films) and PCR as described (*5*). Chloroquine resistance was ruled out for patients if no parasites were detected by microscopy on day 28 or, in case of recurrence, if the chloroquine blood concentration on the day of recurrence did not exceed >100 ng/mL (*6**,**7*). We measured chloroquine blood concentrations by using liquid chromatography–tandem mass spectrometry for 50-μL samples of whole blood.

During January–March 2016, we conducted an additional study at the same site to evaluate the infectivity of *P. vivax* from blood of symptomatic patients to *Anopheles dirus* mosquito vectors; we tested pretreatment and posttreatment blood samples by using membrane feeding assays without serum replacement (*8*). Any febrile patients seeking antimalarial treatment with similar inclusion/exclusion criteria described previously were enrolled in this study.

After we obtained written informed consent, we fed batches of 50 *An. dirus* mosquitoes with blood collected from these patients on 3 occasions: 1) before the first dose of DHA/PPQ or chloroquine, 2) on the same day at 9:00 pm (i.e., 2–11 h after treatment), and 3) at 24h posttreatment for patients treated with chloroquine. We performed statistical analyses by using GraphPad Prism 5 (GraphPad, San Diego, CA, USA) and R software (*9*). Both studies were approved by the Cambodian National Ethic Committee (038 NECHR, 2/24/2014 and 475 NECHR, 12/28/2015).

For the drug comparison study, we enrolled 50 patients (25 in each study arm); in each arm, 5 patients were lost to follow-up during days 2–35. A total of 40 patients (20 in each study arm) were followed up until day 63. Baseline patient characteristics were similar for both patient groups (Table 1). We did not observe any adverse events or early clinical failures. The proportion of patients still parasitemic on days 1 and 2 (detected by microscopy) was lower for the DHA/PPQ–treated group than for the chloroquine-treated group (Table 2). Medians of the parasite reduction ratio recorded on days 1 and 2 were higher for the DHA/PPQ–treated patient group (Table 2). All patients, regardless of their treatment, were microscopically parasite free at day 3.

**Table 1:** Baseline characteristics of *Plasmodium vivax*–infected patients in a clinical drug trial and a human-to-mosquito transmission study, Cambodia*

Characteristic	Chloroquine	DHA/PPQ	p value
Clinical drug trial study, June–December 2014			
No. patients followed up until day 63 (% Male)	20 (80)	20 (80)	1.00†
Patient age, y	26.5 (18.5–35)	28.5 (21.5–46)	0.11‡
Patient weight, kg	56.0 (50–59)	51.0 (49.5–53)	0.14‡
Parasites/μL of blood	5,000 (1,850–8,350)	8,900 (3,500–17,500)	0.051‡
Gametocytes/µL of blood	108 (58–200)	245 (105–745)	0.12‡
Proportion with G6PD deficiency by spot test and PCR§	2/20 (Viangchan variant)	1/20 (Canton variant)	1.00†
Leukocytes, ×10^9 ^cells/L	7.7 (6.2–9.2)	6.9 (5.2–8.6)	0.38‡
Erythrocytes, ×10^12 ^cells/L	5.01 (4.53–5.26)	5.10 (4.69–5.65)	0.42‡
Hemoglobin, g/dL	11.2 (10.3–13.8)	12.3 (11.4–13.1)	0.43‡
Hematocrit, %	37 (34–43)	40 (37–43)	0.49‡
Human-to-mosquito transmission study, January–March 2016¶			
No. (%) male patients	9 (100)	10 (70)	0.21†
Patient age, y	13.0 (12.7–38.5)	24.5 (19.0–29.0)	0.68‡
Patient weight, kg	37.0 (28.7–52.2)	53.5 (42.0–60.0)	0.09‡
Parasites/μL of blood	4,565 (3,462–6,184)	9,069 (6,833–11,591)	**0.01‡**
Gametocytes/μL of blood	221 (74.2–381.5)	1,915 (693–2,729)	**0.001‡**
Proportion of infectious patients before treatment; feeding assay before first dose of treatment#	8/9 (89)	9/10 (90)	1.00†
Proportion of infected mosquitoes before treatment; feeding assay before first dose of treatment	69.6 (26.2–84.9)	72.9 (29.75–92.7)	0.71‡
Average no. oocysts in infected mosquitoes before treatment; feeding assay before first dose of treatment	12.2 (2.4–29.8)	12.2 (2.4–40.6)	0.74‡

*Values are median (IQR) or no. positive/no. tested (%) unless otherwise indicated. Bold indicates statistical significance (p<0.05). DHA/PPQ, dihydroartemisinin/piperaquine; G6PD, glucose-6-phosphate dehydrogenase; IQR, interquartile range. †By Ficher exact test. ‡By Mann-Whitney U test.  §For details, see Khim et al. (*10*). ¶Mosquitoes were 6–8 days old and were allowed to feed for 20 min. Feeding was conducted at the same place immediately after obtaining blood from patients. Dissections were performed 6 days after the blood meal. Midguts were dissected in 1% mercurochrome stain, and the presence and number of oocysts were determined by microscopy (×20 magnification). #Patients were defined as being infectious when >1 mosquito became infected with oocysts.

**Table 2:** *Plasmodium vivax* clearance among infected patients, time to malaria recurrence, and vector transmission results, by allocated antimalarial drug treatment, Cambodia*

Characteristic	Chloroquine	DHA/PPQ	p value
Clinical drug trial study, June–December 2014			
Proportion of patients parasitemic at day 1 by microscopy	17/20 (85)	6/20 (30)	**0.001†**
Parasites/μL of blood at day 1	180 (80–600)	0 (0–57)	**0.0002‡**
Parasite reduction ratio at day	96.2 (68.5–98.6)	100 (99.4–100)	**0.0002‡**
Proportion of patients parasitemic at day 2 by microscopy	5/20 (25)	0/20 (0)	**0.047†**
Parasites/μL of blood at day 2	0 (0–15)	0	**0.03‡**
Parasite reduction ratio at day 2	100 (99.8–100)	100	**0.03‡**
Proportion of patients parasitemic at day 3 by microscopy	0/20 (0)	0/20 (0)	1.00†
Proportion of patients with recurrence detected by PCR	12/20 (60)	4/20 (20)	**0.02†**
Time to recurrence, d	49 (42–49)	56 (52.5–56)	**0.04‡**
Human-to-mosquito transmission study, January–March 2016			
Proportion of infectious patients after first dose of treatment; feeding assay at 9:00 pm	8/9 (89)	1/10 (10)	**0.001†**
Proportion of infected mosquitoes after first dose of treatment; feeding assay at 9:00 pm	60.7 (23.7–78.6)	0	**0.004‡**
Average no. of oocysts in infected mosquitoes after first dose of treatment; feeding assay at 9:00 pm	9.9 (4.1–25.7)	356.4	0.22‡
Parasite transmissibility reduction ratio (%) at 9:00 pm	19 (−13.8 to 62.7)	100	**0.003‡**
Proportion of infectious patient 24 h after first dose of chloroquine	2/9 (22)	ND	ND
Average no. of oocysts in infected mosquitoes after first dose of treatment for 2 infectious patients; feeding assay 24 h after first dose of chloroquine	1.3 and 2.4	ND	ND
Proportion of infected mosquitoes 24 h after first dose of chloroquine	0 (0–4.4)	ND	ND

*Values are no. positive/no. tested (%) or median (IQR) unless otherwise indicated. Bold indicates statistical significance (p<0.05). DHA/PPQ, dihydroartemisinin/piperaquine; IQR, interquartile range; ND, no data. †By Fisher exact test. ‡By Mann-Whitney U test.

**Figure 1:**
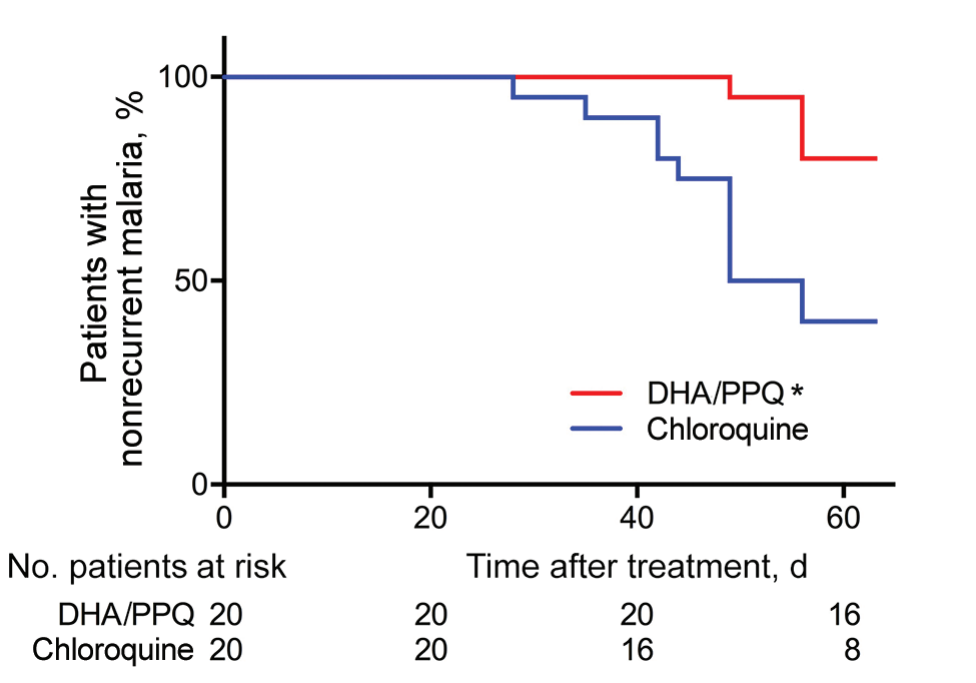
Cumulative proportion of patients with nonrecurrent *Plasmodium vivax* malaria given a 3-day course of DHA/PPQ and chloroquine detected by PCR within 63 days of follow up, Cambodia. *p<0.01, by log-rank test during Kaplan-Meier survival analysis. DHA/PPQ, dihydroartemisinin/piperaquine.

Within 2 months of follow-up, there were fewer patients with a recurrence (detected by PCR) in the DHA/PPQ-treated group than in the chloroquine-treated group (odds ratio 0.17, 95% CI 0.05–0.66; p<0.05, by log-rank test, p<0.01 by Kaplan-Meier survival analysis) (Table 2; Figure 1). Median time to recurrence after treatments was also delayed in patients given DHA/PPQ (56 days) compared with those patients given chloroquine (49 days) (Table 2). No recurrence occurred before day 28 in either study arm, which is suggestive of relapse or reinfection, rather than recrudescence of drug-resistant parasites (*6**,**7*). In the chloroquine-treated group, 12/20 patients with recurrence had a chloroquine blood concentration <100 ng/mL on the day of recurrence (chloroquine + desethyl chloroquine: median 55.6 ng/mL, interquartile range 40.0–61.7 ng/mL); these results excluded likely chloroquine resistance (*6**,**7*).

**Figure 2:**
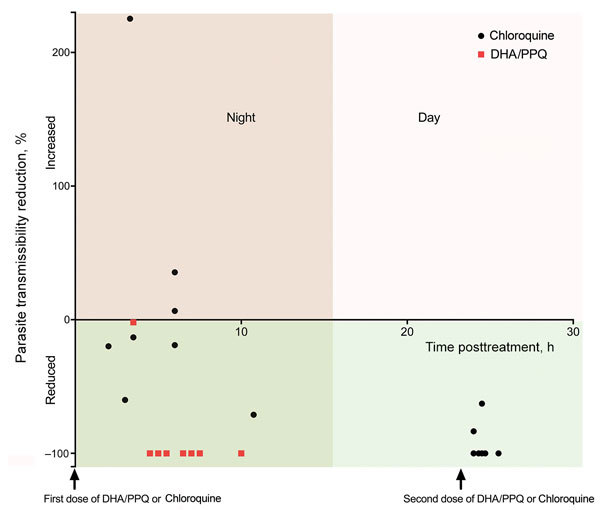
Transmission-blocking efficacy of allocated antimalarial drug treatment (chloroquine and DHA/PPQ) on human-to-mosquito transmission of *Plasmodium vivax*, January–March 2016, Cambodia. Each dot represents the parasite transmissibility reduction ratio (i.e., 100 – [proportion of infected mosquitoes fed with blood samples collected at 9:00 pm after the first dose of treatment × 100/proportion of infected mosquitoes fed with blood samples collected at patient enrollment before the first dose of treatment]). Only data of infectious patients at enrollment are shown (17/19 patients; Table 1). For the chloroquine-treated patient group, a second mosquito blood feeding was performed 24 h after the first dose. DHA/PPQ, dihydroartemisinin/piperaquine.

For the mosquito-to-human transmission study, we enrolled 19 patients (9 given chloroquine and 10 given DHA/PPQ). Baseline patient characteristics were similar in both patient groups, except for day 0 parasitemia and gametocytemia, which were higher for the DHA/PPQ-treated group (Table 1). The proportion of infectious blood from *P. vivax*–infected patients and the median proportion of infected mosquitoes fed on blood collected before the first dose of DHA/PPQ or chloroquine were similar for both patient groups (Table 1). Despite an initial higher day 0 gametocytemia for the DHA/PPQ-treated group, the proportions of infectious *P. vivax* blood collected at 9:00 pm after the first dose of DHA/PPQ or chloroquine and the median proportion of infected mosquitoes were lower for the DHA/PPQ-treated group than for the chloroquine-treated group (Table 2). Overall, DHA/PPQ acted faster than chloroquine in decreasing over time the proportion of infectious patients (generalized linear mixed model time for drug interaction, χ_1_^2^ = 113.1, p< 0.0001) (Figure 2). For the group given chloroquine, 2 (22%) of 9 blood samples were still infectious 24 hours after the first dose (Table 2).

## Conclusions

We confirm that DHA/PPQ acts faster (<48 h) than chloroquine (≈72 h) in eliminating sexual and asexual *P. vivax* parasites and that DHA/PPQ provides an excellent postexposure prophylaxis against potential recurrences for >2 months (*11*). This benefit relies on the combination of artemisinin derivatives (DHA), which are fast-acting drugs capable of eliminating any *P. vivax* blood stages, and a long-lasting partner drug (PPQ), which has a long terminal elimination half-life and is highly effective in preventing *P. vivax* recurrence for up to 56 days. Although the number of patients enrolled was small, we demonstrated that DHA/PPQ also acts faster (<5 h) than chloroquine in killing *P. vivax* sexual stages and thus prevents the risk for transmission of parasites to the mosquito vector the night after uptake of the first dose. This rapid clearance of gametocytes is a major benefit of DHA/PPQ in comparison with chloroquine, given that *P. vivax* gametocytes appear early in the course of disease and must be eliminated as soon as possible to limit risk of transmission (*12**,**13*).

In summary, our findings support the recommendation of DHA/PPQ as first-line treatment for *P. falciparum* and *P. vivax* uncomplicated malaria in regions to which these species are coendemic. These findings apply to areas in which chloroquine is still effective and no *P. falciparum* resistance to PPQ has been observed.

## References

[1] Battle KE, Gething PW, Elyazar IR, Moyes CL, Sinka ME, Howes RE, et al. The global public health significance of *Plasmodium vivax.* Adv Parasitol. 2012;80:1–111. 10.1016/B978-0-12-397900-1.00001-3 23199486

[2] Gething PW, Elyazar IR, Moyes CL, Smith DL, Battle KE, Guerra CA, et al. A long neglected world malaria map: *Plasmodium vivax* endemicity in 2010. PLoS Negl Trop Dis. 2012;6:e1814. 10.1371/journal.pntd.0001814 22970336 PMC3435256

[3] Siv S, Roca-Feltrer A, Vinjamuri SB, Bouth DM, Lek D, Rashid MA, et al. *Plasmodium vivax* Malaria in Cambodia. Am J Trop Med Hyg. 2016;95(Suppl):97–107. 10.4269/ajtmh.16-0208 27708187 PMC5201228

[4] Leang R, Barrette A, Bouth DM, Menard D, Abdur R, Duong S, et al. Efficacy of dihydroartemisinin-piperaquine for treatment of uncomplicated *Plasmodium falciparum* and *Plasmodium vivax* in Cambodia, 2008 to 2010. Antimicrob Agents Chemother. 2013;57:818–26. 10.1128/AAC.00686-12 23208711 PMC3553743

[5] Canier L, Khim N, Kim S, Sluydts V, Heng S, Dourng D, et al. An innovative tool for moving malaria PCR detection of parasite reservoir into the field. Malar J. 2013;12:405. 10.1186/1475-2875-12-405 24206649 PMC3829804

[6] Baird JK, Leksana B, Masbar S, Fryauff DJ, Sutanihardja MA, Suradi, et al. Diagnosis of resistance to chloroquine by *Plasmodium vivax*: timing of recurrence and whole blood chloroquine levels. Am J Trop Med Hyg. 1997;56:621–6. 10.4269/ajtmh.1997.56.621 9230792

[7] World Health Organization. Methods for surveillance of antimalarial drug efficacy, 2009 [cited 2018 Mar 27]. http://apps.who.int/iris/handle/10665/44048

[8] Bousema T, Dinglasan RR, Morlais I, Gouagna LC, van Warmerdam T, Awono-Ambene PH, et al. Mosquito feeding assays to determine the infectiousness of naturally infected *Plasmodium falciparum* gametocyte carriers. PLoS One. 2012;7:e42821. 10.1371/journal.pone.0042821 22936993 PMC3425579

[9] R Development Core Team. R: a language and environment for statistical computing. Vienna: R Foundation for Statistical Computing; 2013.

[10] Khim N, Benedet C, Kim S, Kheng S, Siv S, Leang R, et al. G6PD deficiency in *Plasmodium falciparum* and *Plasmodium vivax* malaria-infected Cambodian patients. Malar J. 2013;12:171. 10.1186/1475-2875-12-171 23714236 PMC3671135

[11] Sinclair D, Gogtay N, Brand F, Olliaro P. Artemisinin-based combination therapy for treating uncomplicated *Plasmodium vivax* malaria. Cochrane Database Syst Rev. 2011; (7):CD008492. 21735431 10.1002/14651858.CD008492.pub2

[12] Sagara I, Beavogui AH, Zongo I, Soulama I, Borghini-Fuhrer I, Fofana B, et al. Safety and efficacy of re-treatments with pyronaridine-artesunate in African patients with malaria: a substudy of the WANECAM randomised trial. Lancet Infect Dis. 2016;16:189–98. 10.1016/S1473-3099(15)00318-7 26601738 PMC4726763

[13] Sawa P, Shekalaghe SA, Drakeley CJ, Sutherland CJ, Mweresa CK, Baidjoe AY, et al. Malaria transmission after artemether-lumefantrine and dihydroartemisinin-piperaquine: a randomized trial. J Infect Dis. 2013;207:1637–45. 10.1093/infdis/jit077 23468056

